# Drug Interactions in Lenacapavir-Based Long-Acting Antiviral Combinations

**DOI:** 10.3390/v14061202

**Published:** 2022-05-31

**Authors:** Maria E. Cilento, Yee Tsuey Ong, Philip R. Tedbury, Stefan G. Sarafianos

**Affiliations:** Laboratory of Biochemical Pharmacology, Department of Pediatrics, Emory University School of Medicine, Children’s Healthcare of Atlanta, Atlanta, GA 30307, USA; maria.cilento@emory.edu (M.E.C.); yee.tsuey.ong@emory.edu (Y.T.O.); philip.tedbury@emory.edu (P.R.T.)

**Keywords:** EFdA, islatravir, synergy, long-acting regimens

## Abstract

Long-acting (LA) anti-HIV regimens show promise for increasing dosing intervals and consequently, improving the patients’ quality of life. The first FDA-approved LA therapy is Cabenuva, which comprises rilpivirine (a non-nucleoside reverse transcriptase inhibitor) and cabotegravir (integrase strand transfer inhibitor). Novel promising LA anti-HIV agents such as lenacapavir (a capsid-targeting antiviral) and islatravir (EFdA, a nucleoside reverse transcriptase translocation inhibitor) need to be explored as combination therapies. Therefore, we sought to determine whether combination of lenacapavir with islatravir, rilpivirine, or cabotegravir displayed synergy, additivity, or antagonism. We performed dose-response matrices of these drug combinations in an HIV-1 reporter cell line and subsequently analyzed the data with SynergyFinder Plus, which employs four major drug interaction models: highest single agent, Bliss independence, Loewe additivity, and zero interaction potency. Most of these models predict additive inhibition by the studied drug combinations This work highlights the importance of effective drug combinations in LA-regimens.

## 1. Introduction

HIV/AIDS-related cases and deaths have decreased over the past decades due to highly active antiretroviral therapies (HAART) [1,2,3,4]. HAART typically consists of three drugs; two nucleoside or nucleotide reverse transcriptase (RT) inhibitors (NRTIs) and one other drug such as a non-nucleoside reverse transcriptase inhibitor (NNRTI) or an integrase strand transfer inhibitor (INSTI) [4]. Most regimens require once-daily dosing. Interruptions in adherence increase the risk of resistance mutations emerging that can result in HAART failure. Due to these concerns, there has been a focus on anti-HIV-1 drugs with long-acting (LA) potential to increase dosing intervals; this is likely to improve regimen adherence and improve patient quality of life. To date, the selection of drug combinations has been driven by the individual characteristics of specific antivirals. Indeed, the first FDA approved LA therapy is Cabenuva, comprising cabotegravir (CAB, an INSTI) in combination with rilpivirine (RPV, an NNRTI) [5], both of which have the pharmacological properties of potency and stability that make them suitable for use as LA agents. However, there are potential combinations of antivirals from diverse classes that could be considered for LA therapies: these include the capsid-targeting lenacapavir (LEN, GS-6207) [6] and islatravir (ISL, MK-8591, 4′-ethynyl-2-fluoro-2′-deoxyadenosine, or EFdA) [7], a nucleoside RT translocation inhibitor (NRTTI) [7]. Therefore, in addition to the approved NNRTI- and INSTI-based combination regimens, it is important to explore LEN combinations with LA therapies such as CAB, RPV, and ISL as antiviral combinations to evaluate interactions for future regimens. Here we use an HIV-1 reporter cell line to examine whether use of LEN with either ISL, CAB, or RPV results in synergy, additivity, or antagonism. These results provide useful information on combinations of various classes of antivirals with LA potential.

## 2. Materials and Methods

HEK-293/17 [8] and TZM-GFP cells [9] (Massimo Pizzato) were cultured in DMEM (Corning, NY, USA), 10% FBS, 2mM L-glutamine (Thermo Fisher, MA, USA), and 100 U/mL penicillin/100 µg/mL streptomycin (Thermo Fisher). 

Virus stock was prepared by transfecting HEK-293/17 cells with 5 μg of NL4-3 using Xtreme-GENE HP (Roche, Switzerland). After 48 h, supernatant was collected, filtered, and concentrated using Lenti-X (Clontech, CA, USA).

TZM-green fluorescent protein (GFP) cells were plated in 96-well plates with serially diluted ISL (Life Chemicals, Niagara-on-the-Lake, ON, Canada), CAB (Selleck Chemicals, Houston, TX, USA), RPV (AIDS Reagent Program, NIH, Germantown, MD, USA), and LEN (Medchem Express, Monmouth Junction, NJ, USA) starting at 5 nM LEN or 20 nM ISL, CAB, and RPV. The cells and antivirals were incubated for 24 h. Next, the cells were infected with virus mixed with DEAE-dextran (1 μg/mL final concentration), and incubated for 48 h. The GFP-positive cells were then imaged using Cytation 5 (BioTek, Winooski, VT, USA) and counted using Gen5 v3.03 (BioTek). For experiments with CAB, luciferase rather than GFP was employed as the readout; after 48 h incubation, cells were lysed with britelite plus (PerkinElmer, MA, USA) and read on GloMax Navigator (Promega, Madison, WI, USA). Dose-response data were normalized to positive and negative controls to determine percent inhibition, then analyzed using SynergyFinder Plus [10]. Negative inhibition values were corrected using SynergyFinder Plus.

There are multiple models used to assess synergy and they have primarily been used to analyze interactions of anti-cancer drugs [10,11,12]. Analysis of the same experimental data using models that operate under distinct null hypotheses of non-interaction, can lead to different interpretations of synergy between tested drugs. To evaluate interactions between antiviral combinations as reliably as possible, we analyzed the data from combination studies using the SynergyFinder Plus calculator that evaluates synergy with four majorly used models: ZIP, LOEWE, BLISS, and HSA. These models were formulated with specific biological or empirical assumptions. ZIP calculates the effect of a two drug combination assuming that each does not affect the potency of the other [12]. The BLISS model assumes a stochastic process where the drugs act independently, while the expected combination is calculated on the probability of independent events [13]. LOEWE considers dose-response of the individual drugs and calculates the expected additive response and if the response is higher than expected, it is denoted as synergy [14]. HSA defines synergy as a combination that gives a greater response than either of the individual drug [15]. The HSA model, unlike the other three, is primarily used in situations where no single agent can achieve 100% response (e.g., many cancer therapeutics), where a response above what can be achieved with a single agent may be regarded as synergy. In contrast, all the anti-virals used in this study can individually achieve 100% inhibition. SynergyFinder Plus calculates a mean of the entire matrix, to determine a synergy score and a *p*-value.

## 3. Results

**Figure 1:**
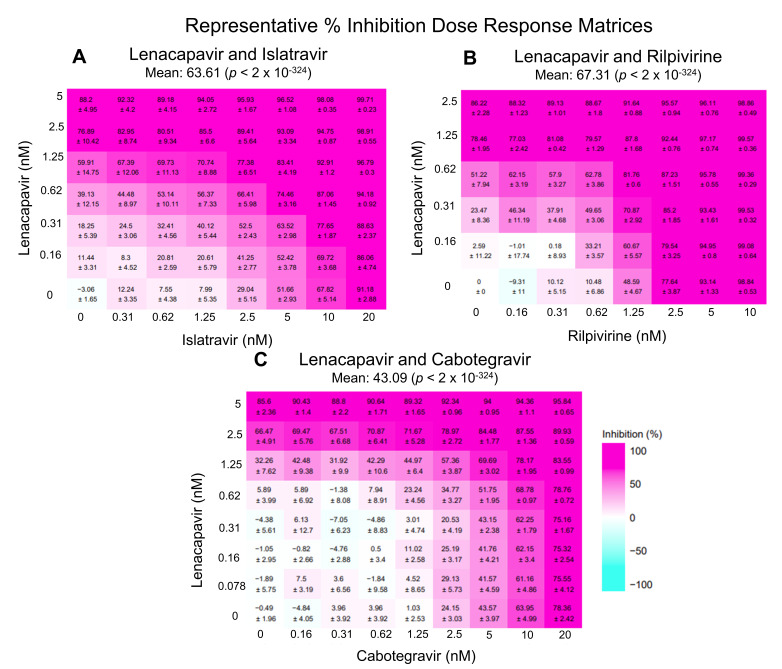
Representative percent inhibition dose response matrix of LEN with ISL, RPV, or CAB. Above demonstrates representative matrices (made with SynergyFinder Plus) of various percent inhibition at each individual dose-response point as well as the overall percent inhibition with the combinations studied: (**A**) LEN and ISL, (**B**) LEN and RPV, and (**C**) LEN and CAB. All negative values are corrected for in SynergyFinder Plus.

To thoroughly evaluate interactions between antivirals we analyzed the data from combination studies using SynergyFinder Plus, a calculator that evaluates synergy applying the four most used models: Zero interaction policy (ZIP), Loewe additivity (LOEWE), Bliss independence (BLISS), and highest single agent (HSA). The different models operate under distinct assumptions, presented in Discussion. *p*-values are also determined by comparing the experimental data to the null hypothesis, a synergy score of zero. While mean score values near zero are interpreted as evidence for additive interactions, a +5 or higher value is considered synergistic, and a −5 or lower antagonistic, when combined with a significant *p*-value [11]. To determine whether LEN was synergistic with ISL, CAB, or RPV, we performed a matrix of dose-response curves with these drug combinations (Figure 1). 

### 3.1. LEN-ISL

**Figure 2:**
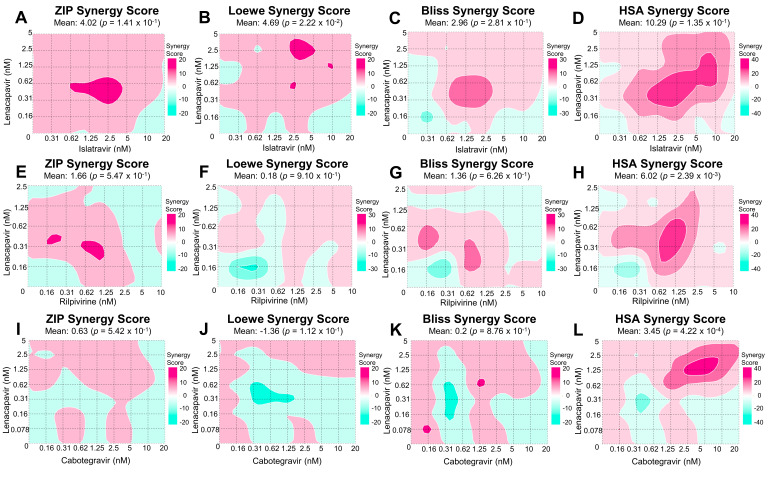
Synergy scores and matrices of LEN and ISL, RPV, or CAB. Above demonstrates the various synergy scores at each individual dose-response point, as well as the overall synergy score for ZIP, LOEWE, BLISS, and HSA. Each row represents different drug combinations: (**A***–***D**) LEN and ISL, (**E***–***H**) LEN and RPV, and (**I***–***L**) LEN and CAB. Each drug combination was performed in TZM-GFP cells, where dose-response matrices were pre-incubated 24 h with the cells. After, cells were incubated with NL4-3 and 48 h post infection the cells were imaged for GFP (**A***–***H**). Cells treated with LEN and CAB were lysed and read for luciferase signal (**I***–***L**). Furthermore, percent inhibition was calculated (as seen in Figure 1), and then input into SynergyFinder Plus, where these 2D contour plots, synergy scores, and mean synergy scores were calculated.

LEN and ISL target HIV-1 capsid and the polymerase function of RT, respectively. The mean values of ZIP, BLISS, and LOEWE synergy scores appeared to be additive at 4.02, 2.96, and 4.69, respectively (Figure 2A–C). The ZIP and BLISS models did not deviate significantly from the predicted no-interaction response (*p* = 0.141 and 0.281). In contrast, the LOEWE model reported significant deviation from the predicted no-interaction response (*p* = 0.022), even though the magnitude of the effect was slightly below the cut-off to assign synergy. Analysis through the HSA model reported statistically significant synergy (synergy score = 10.3; *p* = 0.0001) (Figure 2D). Taken together, these data suggest that these inhibitors likely act through an additive or mildly synergistic mode. 

### 3.2. LEN-RPV

LEN and RPV block HIV-1 by binding the capsid and NNRTI binding region of RT, respectively. Similarly, to the LEN-ISL combination, the ZIP, LOEWE, and BLISS synergy scores suggested additivity (1.66, 0.18, and 1.36, respectively; Figure 2E–G) and the values did not suggest significant deviation from the no-interaction prediction (*p* = 0.547, 0.91, and 0.626, respectively). HSA-based analysis suggested a marginally synergistic, statistically significant score (6.02, *p* = 0.00239 in Figure 2H). Collectively, these data suggest that these inhibitors likely act through an additive or marginally synergistic mode.

### 3.3. LEN-CAB

Data in Figure 2I–L show that combination of a capsid-targeting compound, LEN, and an INSTI, CAB, result in additive inhibition of HIV-1, as suggested by the scores estimated by all four models (the ZIP, LOEWE, BLISS, and HSA synergy mean scores were 0.63, −1.36, 0.2, and 3.45, respectively). The scores for the ZIP, LOEWE, and BLISS were not statistically significant (*p* = 0.542, 0.112, and 0.876, respectively), while the HSA model’s *p*-value was 0.00042.

## 4. Discussion

LEN and ISL were only overall synergistic with the HSA model, but the shortcoming of this model is these inhibitors reach 100% HIV-1 inhibition. Furthermore, the BLISS model was found to be additive, and we were unable to reject the null hypothesis of no-interaction. The data were also consistent with the no-interaction responses predicted by the LOEWE and ZIP models, suggesting additive responses. When all four models and the entire matrix are taken into account, these two drugs show additivity. Of note, higher dose combinations of ISL and LEN were synergistic in all four models, and this could be further explored in other experimental model systems. LEN and RPV were found to be overall additive using ZIP, LOEWE, and BLISS. The HSA model reported synergy. When taking all results into account, these two drugs are most likely additive. We did not observe consistent synergy between LEN and RT-targeting compounds, even though at high concentrations capsid-targeting compounds can inhibit reverse transcription. Finally, LEN and CAB were also seen to be additive in all models (ZIP, LOEWE, HSA, and BLISS), which supports a conclusion of additivity. 

The potential of anti-HIV drugs to be used in long-acting formulations will be important for the future of HAART, to increase patient adherence and decrease the emergence of viral breakthrough resistance mutations. In the combinations of the drugs studied: LEN and ISL, RPV, or CAB, we found primarily additive interactions. The lack of antagonism is encouraging for future use of these agents in long-acting combinations. These assays could be used for preliminary screening of drug interactions between compounds. Synergy could be further evaluated in animal models, which account for more mechanisms of drug interaction. These may include differences in metabolic activation or clearance of individual drugs, as well as changes in their pharmacological characteristics in the context of combinations. In addition, metabolic characteristics may also be affected by the use of agents such as ritonavir, which may not directly enhance in vitro efficacy. Another factor that affects the effectiveness of combination therapies is the genetic barrier to resistance of drugs used individually or in combinations. Such factors are not effectively modeled in single round in vitro experiments but have great value in vivo during prolonged therapeutic use. In conclusion, the present study highlights that these drugs hold considerable promise for combination use in LA-regimens.

## Data Availability

Not applicable.
